# Account of Diseases in an Eastern District of London, from the 20th of November to the 20th of December, 1800

**Published:** 1801-01

**Authors:** 


*Account of Diſeaſes in an Eaſtern Diſtrict of London, from the 20th of November to the 20th of December,* 1800.

| | No. of Caſes. |
|---|---|
| ACUTE DISEASES. | |
| Typhus - - - - - - - - | 15 |
| Sore Throat - - - - - | 3 |
| Peripneumony - - - - - | 2 |
| Dyſenteria - - - - - - | 1 |
| Small Pox - - - - - - | 3 |
| CHRONIC DISEASES. | |
| Cough - - - - - - - | 18 |
| Cough with Dyſpnœa - | 17 |
| Dyſpnœa - - - - - - | 12 |
| Hæmoptyſis - - - - - | 3 |
| Catarrh - - - - - - - | 7 |
| Phthiſis Pulmonalis - - | 4 |
| Gaſtrodynia - - - - - | 8 |
| Hæmatemeſis - - - - - | 1 |
| Dyſpepſia - - - - - - | 7 |
| Diarrhœa - - - - - - | 10 |
| Enterodynia - - - - - | 4 |
| Colica - - - - - - - | 2 |
| Epilepſia - - - - - - | 2 |
| Hyſteria - - - - - - - | 6 |
| Apoplexy - - - - - - | 1 |
| Hemiplegia - - - - - - | 2 |
| Hypochondriaſis - - - - | 3 |
| Chloroſis - - - - - - | 5 |
| Fluor Albus - - - - - - | 8 |
| Amenorrhœa - - - - - | 7 |
| Chronic Eruptions - - | 15 |
| Lumbago - - - - - - | 2 |
| Chronic Rheumatiſm - - | 20 |
| PUERPERAL DISEASES. | |
| Ephemera - - - - - - | 4 |
| Milk Fever - - - - - - | 2 |
| Menorrhagia Lochialis - | 3 |
| Maſtodynia - - - - - - | 7 |
| INFANTILE DISEASES. | |
| Aphthæ - - - - - - - | 3 |
| Convulſio - - - - - - | 4 |
| Ophthalmia - - - - - - | 2 |
| Ophthalmia Purulenta - | 4 |

The fever, which a few weeks ago was confidered as the prevailing epidemic, though the violence of its fymptoms has abated, ftill continues to engage the attention of medical men. Violent affections of the head, the fymptom by which it was particularly characterized at its firft appearance, occur lefs frequently; but the inflammation and flight ulcerations of the throat, which were taken notice of in the laft report, ftill continue very frequently to attend the other fymptoms.

We have now entered upon that feafon of the year in which affections of the lungs form a very confiderable proportion in a lift of difeafes. Cough and dyfpnœa, inflammation of the lungs, hæmoptyfis and catarrh, are perpetually requiring medical affiftance.

Thefe complaints, it is well known, prove troublefome and tedious, and, in conftitutions which are fubject to them, very frequently continue in a greater or lefs degree of aggravation through the winter. An early attention to the fymptoms of thefe complaints might, in fome inftances, produce a fpeedy termination: but what is commonly called a cold, is confidered as a difeafe too frequent and too flight to require any particular notice, efpecially amongft perfons engaged in bufinefs, who confider the confinement and abfence from their ufual occupation, as greater evils than the difeafe itfelf: thus it is left to take its chance, till, in fome patients, it degenerates into an inflammation of the lungs, and terminates in a confirmed phthifis pulmonalis.

